# Supplementary material for: Image-based consensus molecular subtyping in rectal cancer biopsies and response to neoadjuvant chemoradiotherapy
Source: NPJ Precis Oncol. 2024 Apr 9;8:89. doi: 10.1038/s41698-024-00580-3 (PMC11003957; doi:10.1038/s41698-024-00580-3)
Supplement: Supplementary file 1 — REPORTING SUMMARY [file 41698_2024_580_MOESM1_ESM.pdf]

nature portfolio

Corresponding author(s): Viktor Koelzer

Last updated by author(s): Feb 7, 2024

Reporting Summary

Nature Portfolio wishes to improve the reproducibility of the work that we publish. This form provides structure for consistency and transparency in reporting. For further information on Nature Portfolio policies, see our Editorial Policies and the Editorial Policy Checklist.

Statistics

For all statistical analyses, confirm that the following items are present in the figure legend, table legend, main text, or Methods section.

n/a	Confirmed	
☐	☒	The exact sample size (*n*) for each experimental group/condition, given as a discrete number and unit of measurement
☐	☒	A statement on whether measurements were taken from distinct samples or whether the same sample was measured repeatedly
☐	☒	The statistical test(s) used AND whether they are one- or two-sided<br>*Only common tests should be described solely by name; describe more complex techniques in the Methods section.*
☐	☒	A description of all covariates tested
☒	☐	A description of any assumptions or corrections, such as tests of normality and adjustment for multiple comparisons
☐	☒	A full description of the statistical parameters including central tendency (e.g. means) or other basic estimates (e.g. regression coefficient) AND variation (e.g. standard deviation) or associated estimates of uncertainty (e.g. confidence intervals)
☐	☒	For null hypothesis testing, the test statistic (e.g. *F*, *t*, *r*) with confidence intervals, effect sizes, degrees of freedom and *P* value noted<br>*Give P values as exact values whenever suitable.*
☒	☐	For Bayesian analysis, information on the choice of priors and Markov chain Monte Carlo settings
☒	☐	For hierarchical and complex designs, identification of the appropriate level for tests and full reporting of outcomes
☒	☐	Estimates of effect sizes (e.g. Cohen's *d*, Pearson's *r*), indicating how they were calculated

Our web collection on statistics for biologists contains articles on many of the points above.

Software and code

Policy information about availability of computer code

Data collection	Whole Slides Images were reviewed for quality control and tumor regions were annotated using the HALO software (v3.6) (Indica Labs).
Data analysis	For the S:CORT cohorts, gene exppression microarray data were normalised using the robust multiarray average normalization of the Affymetrix package (v1.56.0) in R. Batch-corrected transcriptional CMS calls were derived for each sample with CMSclassifier. Statistical analyses were conducted using R (R Core Team (2022). R: A language and environment for statistical computing. R Foundation for Statistical Computing, Vienna, Austria.https://www.r-project.org/). Development of deep learning models and image analysis was conducted using python (v3.8), openslide (v1.1), numpy (v1.19), pytorch (v1.7). The source code of the underlying (trained) models is not available due to proprietary reasons.

For manuscripts utilizing custom algorithms or software that are central to the research but not yet described in published literature, software must be made available to editors and reviewers. We strongly encourage code deposition in a community repository (e.g. GitHub). See the Nature Portfolio guidelines for submitting code & software for further information.

Data

Policy information about availability of data

All manuscripts must include a data availability statement. This statement should provide the following information, where applicable:

- Accession codes, unique identifiers, or web links for publicly available datasets
- A description of any restrictions on data availability
- For clinical datasets or third party data, please ensure that the statement adheres to our policy

The S:CORT datasets analysed in this study are available from the corresponding author upon reasonable request in accordance with the S:CORT data access policy. The TCGA data analysed in this study are openly and publicly available at https://portal.gdc.cancer.gov/.

Research involving human participants, their data, or biological material

Policy information about studies with human participants or human data. See also policy information about sex, gender (identity/presentation), and sexual orientation and race, ethnicity and racism.

Reporting on sex and gender

Information on sex and gender was not used in this study.

Reporting on race, ethnicity, or other socially relevant groupings

Information on race, ethnicity, or other socially relevant groupings was not used in this study.

Population characteristics

To train and validate the deep learning models developed in this study, H&E-stained tissue specimens of diverse stage and clinical settings were used from three cohorts from the Medical Research Council (MRC) and Cancer Research UK (CRUK) Stratification in COloRecTal cancer (S:CORT) programme: FOCUS (colon and rectal resections, n=365 patients; n=704 slides), randomised clinical trial testing different strategies of sequential and combination chemotherapy for patients with advanced CRC after surgical resection (MRC FOCUS, ISRCTN79877428) [1].

SPINAL (colon and rectal resections, n=206 patients; n=410 slides), patients with primary tumors without previous treatment balanced according to T stage, N stage, location (colon/rectum) and recur rence/metastatic disease (either at diagnosis or during follow-up). Samples were obtained from Birmingham and Manchester Hospitals, United Kingdom and the COIN clinical trial (ISRCTN27286448) [2].

GRAMPIAN (rectal preoperative biopsies, n=233 patients; n=414 slides; sequential cohort of high risk RC with threatened or involved circumferential rectal fascia on pre-treatment MRI scan treated at Aberdeen Royal Infirmary, United Kingdom. For investigation with clinical outcomes, we used H&E-stained tissue from the UK national clinical ARISTOTLE trial and a sequential cohort of high-risk RC with threatened or involved circumferential rectal fascia on pre-treatment MRI scan treated at the University Clinic in Salzburg, Austria (SALZBURG). All patients in the ARISTOTLE and SALZBURG cohorts were strictly selected to have undergone the same treatment protocol for advanced rectal cancer by pelvic irradiation combined with single agent fluoropyrimidine.

ARISTOTLE (rectal preoperative biopsies, n=300 patients; n=300 slides, (base cohort)) UK national clinical trial (ISRCTN09351447) [3] which compared the efficacy of standard CRT with (intervention) or without (control) irinotecan in high-risk RC with threatened or involved circumferential rectal fascia on pre-treatment MRI scan. Cases were selected from the control arm of the ARISTOTLE trial and in whom biopsies were available for molecular analysis. Pathological response was assessed centrally according to a pre-specified pathology protocol using the Dworak method (NPW Leeds).

SALZBURG (rectal preoperative biopsies, n=61 patients; n=61 slides); sequential cohort of high risk RC treated at the IIIrd Department of Internal Medicine of the Paracelsus Medical University Salzburg, Salzburg, Austria; Patients received neoadjuvant long-course chemoradiotherapy with single agent capecitabine as detailed in “RC Treatment”. Pathological response was assessed by detailed histopathological assessment of the resection specimen, undertaken 6-12 weeks after CRT using the Dworak method.

Clinical data was anonymized by S:CORT number and was provided comprising demographic data, baseline stage generated from pre-treatment pelvic MRI scans and CT scans TAP, and outcome data. TCGA H&E-stained tissue samples of The Cancer Genome Atlas Colon Adenocarcinoma (TCGA COAD) and Rectal Cancer Rectum Adenocarcinoma (TCGA-READ) data collection (colon and rectal resections, n=430 patients; n=431 slides) described and made available by the TCGA Research Network at https://www.cancer.gov/tcga [4].

[1] M. Seymour, T. Maughan, J. Ledermann et al. Different strategies of sequential and combination chemotherapy for patients with poor prognosis advanced colorectal cancer (MRC FOCUS): a randomised controlled trial. The Lancet, 370(9582):143–152, 2007.
[2] R. Adams, A. Meade, M. Seymour et al. Intermittent versus continuous oxaliplatin and fluoropyrimidine combination chemotherapy for first-line treatment of advanced colorectal cancer: results of the randomised phase 3 MRC COIN trial. The Lancet Oncology, 12(7):642–653, 2011.
[3] D. Sebag-Montefiore, R. Adams, S. Gollins et al. ARISTOTLE: a phase III trial comparing concurrent capecitabine with capecitabine and irinotecan (Ir) chemoradiation as preoperative treatment for MRI-defined locally advanced rectal cancer (LARC). J Clin Oncol, 38:4101–4101, 2020.
[4] Cancer Genome Atlas Network. Comprehensive molecular portraits of human breast tumours. Nature, 490(7418):61–70, 2012.

Recruitment	Cases were selected from existing clinical trial cohorts and population based cohorts as described in [Population characteristics]. No new patients were recruited for this study.
Ethics oversight	All samples in the S:CORT cohorts (FOCUS, SPINAL, GRAMPIAN, ARISTOTLE) were obtained following individual informed consent and ethical approval by the National Research Ethics Service in the United Kingdom (ref 15/EE/0241; IRAS reference 169363). The SALZBURG cohort was reviewed by the ethical board of the provincial government of Salzburg, Austria (415-E/2343/5-2018), although under Austrian law informed consent is not needed for research use and is therefore not available for all cases.

Note that full information on the approval of the study protocol must also be provided in the manuscript.

Field-specific reporting

Please select the one below that is the best fit for your research. If you are not sure, read the appropriate sections before making your selection.

☒ Life sciences ☐ Behavioural & social sciences ☐ Ecological, evolutionary & environmental sciences

For a reference copy of the document with all sections, see nature.com/documents/nr-reporting-summary-flat.pdf

Life sciences study design

All studies must disclose on these points even when the disclosure is negative.

Sample size	No sample-size calculation was performed, all the eligible data points from the collected cohorts were used for this retrospective study.
Data exclusions	Data was excluded based on incomplete data (exclusion of cases with missing images and exclusion of cases with images of poor quality assessed by an expert pathologist). Non-tumor regions in the analysed images were excluded based on annotations made by an expert pathologist. For model training and evaluation, cases with missing or undefined transcriptional CMS classes were excluded. For the analysis of association between imCMS calls and treatment outcome, cases with missing outcome data were excluded.
Replication	Assessment of classification performance of imCMS was systematically reported using data from multiple independent holdout cohorts. Association between imCMS classification and treatment outcome was conducted on a combined dataset from two independent cohorts.
Randomization	No randomization was necessary to complete this retrospective study.<br>In the logistic regression analysis, models were adjusted by "cohort" and the clinical confounders "pretreatment T stage" and "pretreatment N stage".
Blinding	As this is a retrospective study, no group allocation was performed.

Reporting for specific materials, systems and methods

We require information from authors about some types of materials, experimental systems and methods used in many studies. Here, indicate whether each material, system or method listed is relevant to your study. If you are not sure if a list item applies to your research, read the appropriate section before selecting a response.

Materials & experimental systems

n/a	Involved in the study	
☒	☐	Antibodies
☒	☐	Eukaryotic cell lines
☒	☐	Palaeontology and archaeology
☒	☐	Animals and other organisms
☐	☒	Clinical data
☒	☐	Dual use research of concern
☒	☐	Plants

Methods

n/a	Involved in the study	
☒	☐	ChIP-seq
☒	☐	Flow cytometry
☒	☐	MRI-based neuroimaging

Clinical data

Policy information about clinical studies

All manuscripts should comply with the ICMJE guidelines for publication of clinical research and a completed CONSORT checklist must be included with all submissions.

Clinical trial registration	FOCUS: data was collected via the FOCUS clinical trial (MRC FOCUS, ISRCTN79877428).<br>SPINAL: data was collected via the Birmingham and Manchester Hospitals, United Kingdom and the COIN clinical trial (ISRCTN27286448).<br>ARISTOTLE: data was collected via the ARISTOTLE clinical trial (ISRCTN09351447).<br>GRAMPIAN: data was collected via the Aberdeen Royal Infirmary, United Kingdom (hospital-based cohort).

	SALZBURG: data was collected via the IIIrd Department of Internal Medicine of the Paracelsus Medical University Salzburg, Salzburg, Austria (hospital-based cohort).
Study protocol	This is a retrospective study that used whole slide images, transcriptional data and clinical data from the clincal cohorts mentioned above to study the association between a biopsy image-based signature (imCMS) and treatment outcome in rectal cancer patients (pathological complete response to neoadjuvant long course chemoradiotherapy (LCRT) with single agent fluoropyrimidine).<br>The individual study protocols of the related clinical trials can be retrieved with the reference cited above.
Data collection	The individual data collection protocols of the related clinical trials can be retrieved with the reference cited above.
Outcomes	The primary and secondary outcome measures of the related clinical trials can be retrieved with the reference cited above.

Plants

Seed stocks	*Report on the source of all seed stocks or other plant material used. If applicable, state the seed stock centre and catalogue number. If plant specimens were collected from the field, describe the collection location, date and sampling procedures.*
Novel plant genotypes	*Describe the methods by which all novel plant genotypes were produced. This includes those generated by transgenic approaches, gene editing, chemical/radiation-based mutagenesis and hybridization. For transgenic lines, describe the transformation method, the number of independent lines analyzed and the generation upon which experiments were performed. For gene-edited lines, describe the editor used, the endogenous sequence targeted for editing, the targeting guide RNA sequence (if applicable) and how the editor was applied.*
Authentication	*Describe any authentication procedures for each seed stock used or novel genotype generated. Describe any experiments used to assess the effect of a mutation and, where applicable, how potential secondary effects (e.g. second site T-DNA insertions, mosiacism, off-target gene editing) were examined.*
