# Supplementary material for: Image-based consensus molecular subtyping in rectal cancer biopsies and response to neoadjuvant chemoradiotherapy
Source: NPJ Precis Oncol. 2024 Apr 9;8:89. doi: 10.1038/s41698-024-00580-3 (PMC11003957; doi:10.1038/s41698-024-00580-3)
Supplement: Supplementary file 2 — Supplementary Information [file 41698_2024_580_MOESM2_ESM.pdf]

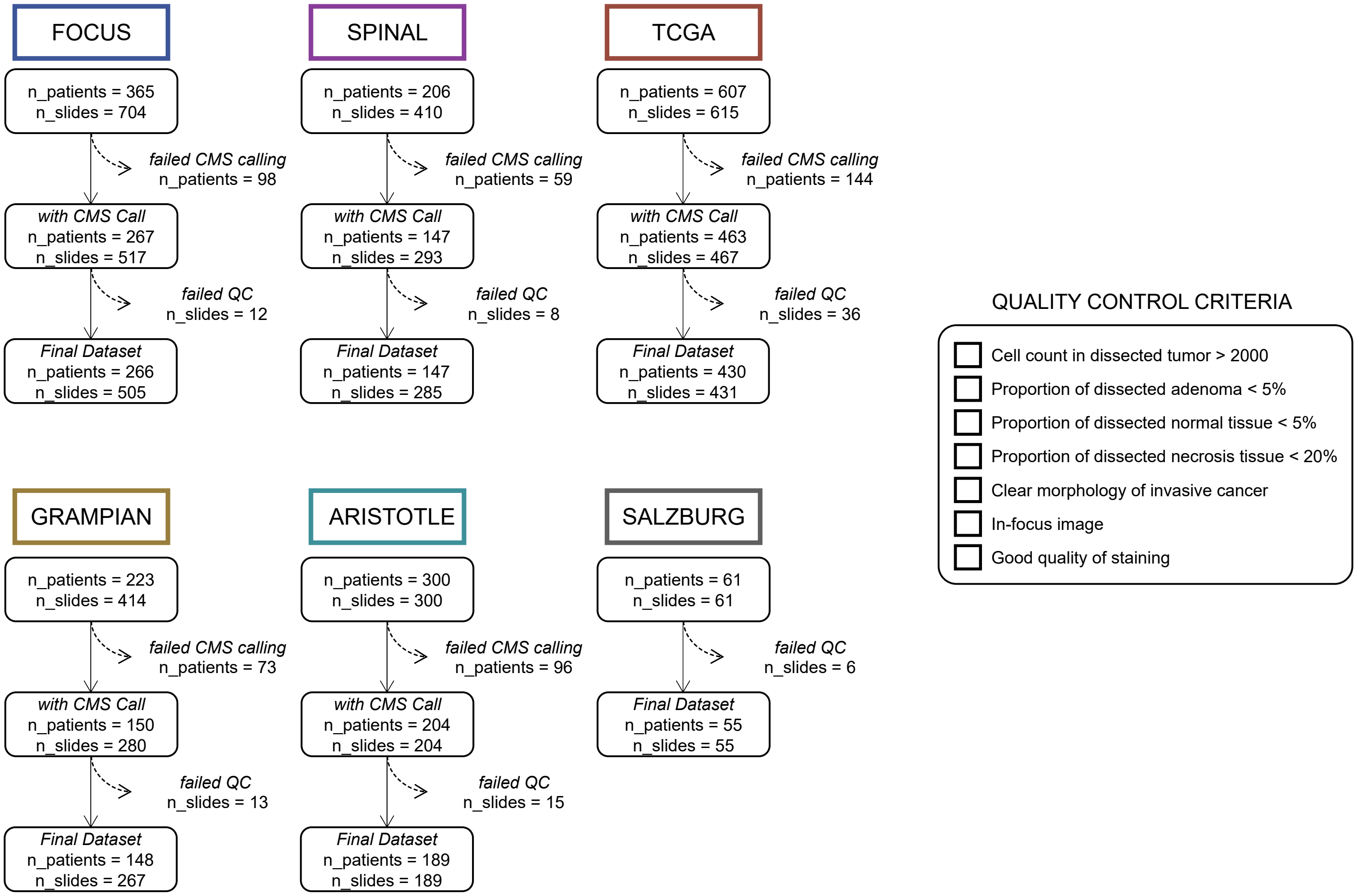

Supplementary Figure 1. Detailed description of the six cohorts used in this study. For each cohort, a flowchart indicates how many patients and images were successively excluded from the study based on the failure of transcriptional CMS calling, and based on visual assessment of the quality of the WSIs using the criteria listed on the right.

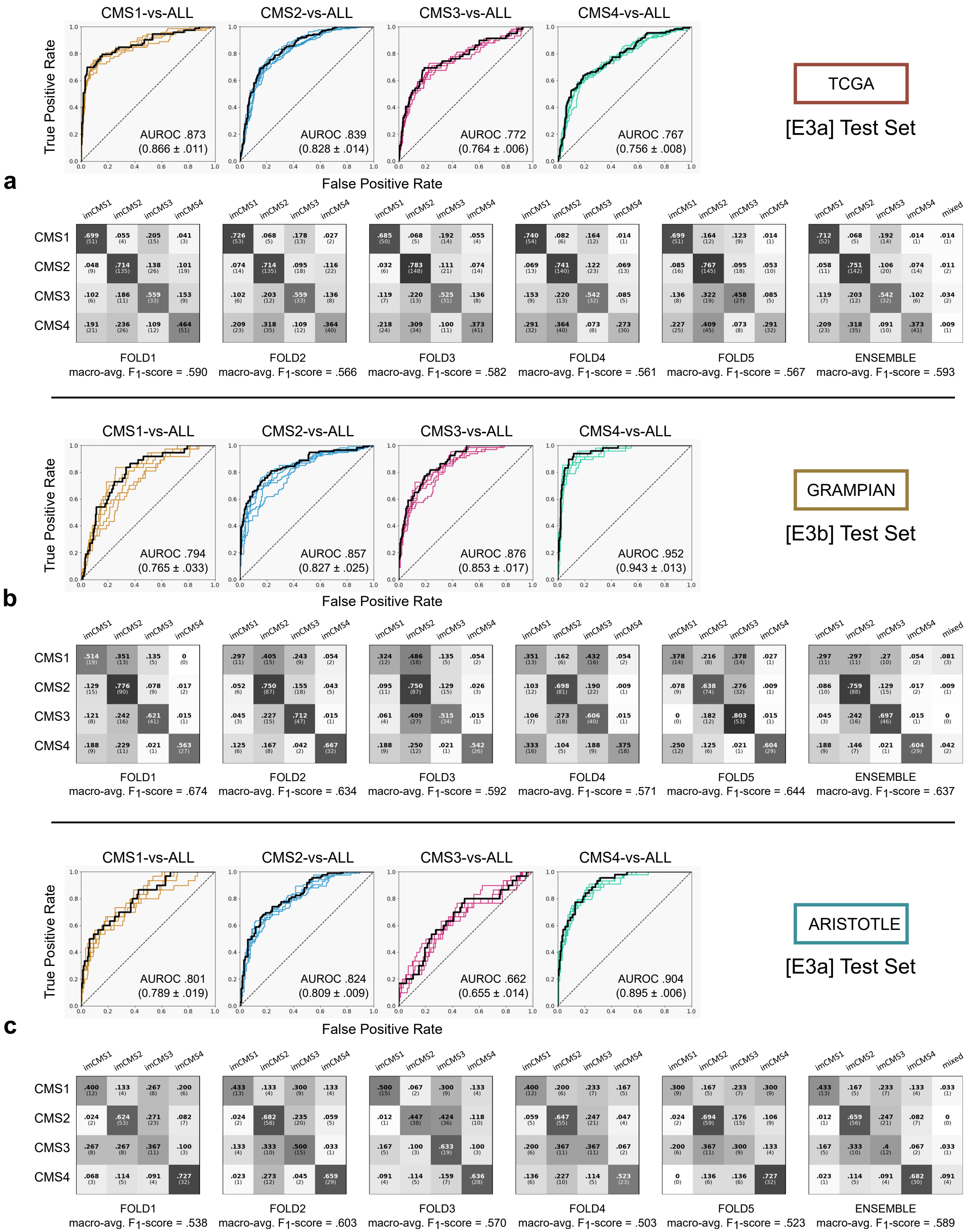

Supplementary Figure 2. Detailed Receiver Operating Curves (ROC) and confusion matrices (CMs) of the trained imCMS models of experiments [E3a] with TCGA (a) and ARISTOLE (c) as test sets, and of experiments [E3b] with GRAMPIAN (b) as test set. Area under the ROC (AUROC) are shown for each CMS class and each of the five trained models of each experiment. Results of majority voting of five models (ensemble model) are reported in the right-most CMs. Cases without absolute majority were classified as "mixed". The reported macro-average F1-scores are derived from the confusion matrices and are ranging from 0 to 1, with 1 indicating perfect classification.

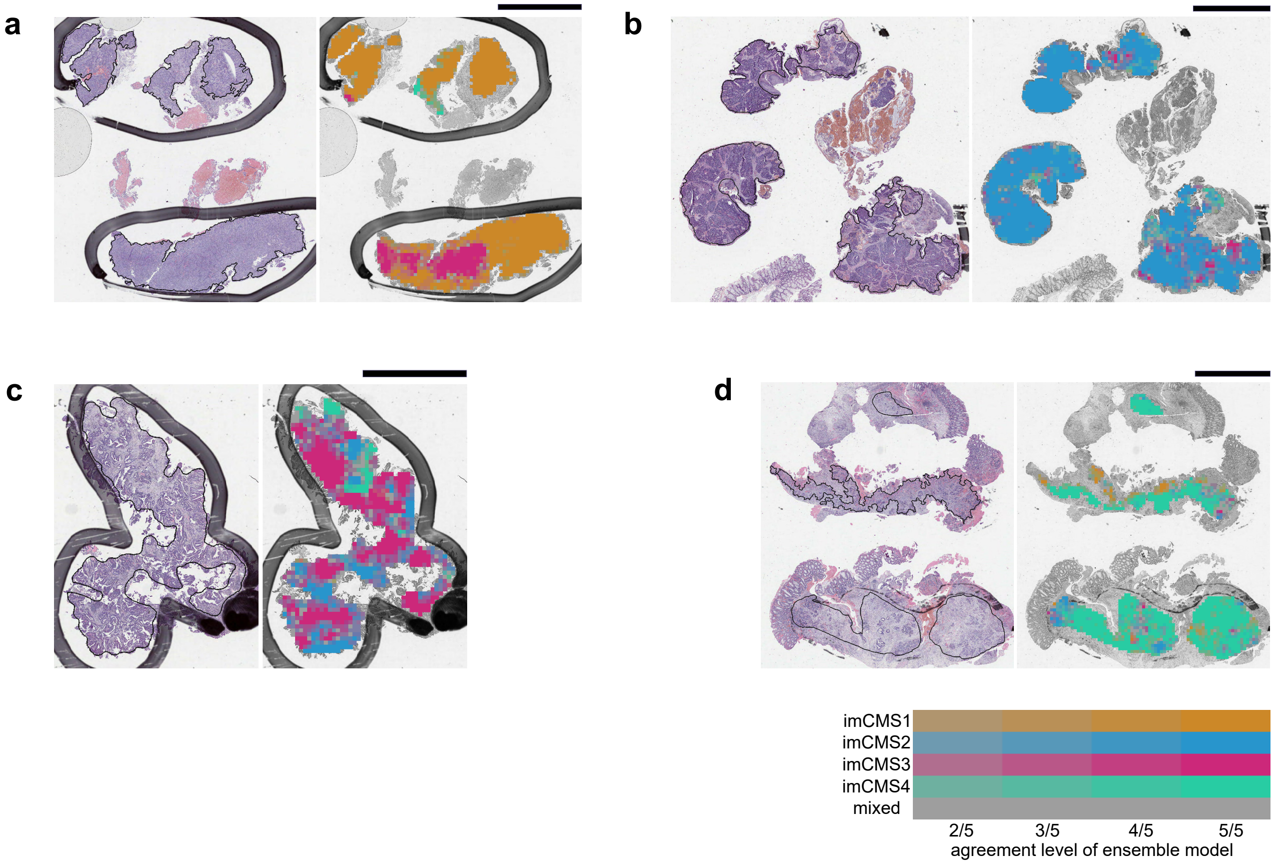

Supplementary Figure 3. Examples of slide-level imCMS classification maps of biopsy specimens. These maps were generated with the ensemble model of experiment [E3a] for correctly classified WSIs of the ARISTOTLE cohort. Pathologist annotation of invasive cancer regions are indicated by black lines (digital) or pen-marks (analogue). Ground-truth transcriptional CMS calls: CMS1 (a); CMS2 (b); CMS3 (c); CMS4 (d). Color visualization is based on the highest voted tile-level predicted imCMS class among the five trained models that form the ensemble model of experiment [E3a] as indicated in the bottom-right legend. Classification maps illustrate the spatially resolved imCMS calls across biopsy samples including samples with some level of pervasive heterogeneity as previously described for both image-based as well as molecular analysis methods [1–3]. Scale bars represent 2mm.

SUPPLEMENTARY NOTES

Additional Details on Clinical Cohorts

To train and validate the deep learning models developed in this study, H&E-stained tissue specimens of diverse stage and clinical settings were used from three cohorts from the Medical Research Council (MRC) and Cancer Research UK (CRUK) Stratification in COloRecTal cancer (S:CORT) programme:

FOCUS (colon and rectal resections, n=365 patients; n=704 slides), randomised clinical trial testing different strategies of sequential and combination chemotherapy for patients with advanced CRC after surgical resection (MRC FOCUS, ISRCTN79877428) [4].

SPINAL (colon and rectal resections, n=206 patients; n=410 slides), patients with primary tumors without previous treatment balanced according to T stage, N stage, location (colon/rectum) and recurrence/metastatic disease (either at diagnosis or during follow-up). Samples were obtained from Birmingham and Manchester Hospitals, United Kingdom and the COIN clinical trial (ISRCTN27286448) [5].

GRAMPIAN (rectal preoperative biopsies, n=233 patients; n=414 slides; sequential cohort of high-risk RC with threatened or involved circumferential rectal fascia on pre-treatment MRI scan treated at Aberdeen Royal Infirmary, United Kingdom. We report a median number of fragments containing tumor content across the biopsies of the GRAMPIAN cohort of 4.

ARISTOTLE (rectal preoperative biopsies, n=300 patients; n=300 slides, (base cohort)) UK national clinical trial (ISRCTN09351447) [6] which compared the efficacy of standard CRT with (intervention) or without (control) irinotecan in high-risk RC with threatened or involved circumferential rectal fascia on pre-treatment MRI scan. Cases were selected from the control arm of the ARISTOTLE trial and in whom biopsies were available for molecular analysis. Pathological response was assessed centrally according to a pre-specified pathology protocol using the Dworak method (NPW Leeds). We report a median number of fragments containing tumor content across the biopsies of the ARISTOTLE cohort of 4.

SALZBURG (rectal preoperative biopsies, n=61 patients; n=61 slides); sequential cohort of high-risk RC treated at the IIIrd Department of Internal Medicine of the Paracelsus Medical University Salzburg, Salzburg, Austria; Patients received neoadjuvant long-course chemoradiotherapy with single agent capecitabine as detailed in "RC Treatment". Pathological response was assessed by detailed histopathological assessment of the resection specimen, undertaken 6-12 weeks after CRT using the Dworak method.

The ARISTOTLE and SALZBURG cohorts were strictly selected to have undergone the same treatment protocol for advanced rectal cancer by pelvic irradiation combined with single agent fluoropyrimidine.

Clinical data was anonymized by S:CORT number and was provided comprising demographic data, baseline stage generated from pre-treatment pelvic MRI scans and CT scans TAP, and outcome data.

TCGA H&E-stained tissue samples of The Cancer Genome Atlas Colon Adenocarcinoma (TCGA-COAD) and Rectal Cancer Rectum Adenocarcinoma (TCGA-READ) data collection (colon and rectal resections) are described and made available by the TCGA Research Network at https://www.cancer.gov/tcga [7].

Transcriptional CMS classification

To obtain robust CMS calls, initially three different CMS classifications were derived from three different transcriptomic datasets. The first transcriptome was composed of each single cohort analyzed independently, the second from a homogenized transcriptomic dataset correcting batch effects by cohort with ComBat and the third from a dataset including all S:CORT cases also batch-corrected by cohort. For the three transcriptomes, gene-level data was obtained with the mean of probe sets linked to each NCBI Entrez Gene ID according to the latest xcel annotation file (v36). Then, CMS calls were derived with the random forest CMSclassifier with the default posterior probability of 0.5. Random forest classification of FFPE samples leads to an increased frequency of unclassified samples compared to published datasets derived from fresh frozen material [1].

To derive calls with lower frequency of unclassified cases, we additionally computed single sample predictor calls after row-centering the expression data. CMS calls were generated when there was a match between both methods (RF and single sample predictor without applying any cut-off) as previously [1]. After obtaining three CMS calls from the three transcriptomes for each sample, a final call for training imCMS was computed when all three CMS calls were matching or when two of them were matching and one was unclassified. This method ensures high quality, robust transcriptomic CMS calls from FFPE tissue.

Deep Learning Model architecture and Training Procedure

The imCMS v1.5 classification model is based on a customized ResNet convolutional neural network [8] with 55 layers that make use of rotation-equivariant convolutional layers as described in [9]. This modification provides guarantees that

the model predictions do not depend on the orientation of the input (for 90-degree rotation angles), which was shown to be beneficial across several classification tasks for histopathology image analysis [9–11]. This architecture was designed to take image patches of size 318×318px (∼636×636 μm^2 at magnification 5×) as input and to output the probability scores (softmax-activated vector of four logit values) for the transcriptional CMS class of the tumor from which image patches originated.

For each training/validation split of the five folds of each investigated multi-cohort development set in this study, we trained a model according to a two-stage procedure. In a first stage, under a weakly supervised framework, each model was trained to predict slide-level CMS classes from tile-level input image patches. Each model was trained via minimization of the cross-entropy loss using random batches of size 64 from the training partition with uniform distribution of transcriptional CMS. Models were trained with gradient descent with initial learning rate 0.01, cycling cosine annealing (period of 10 000 iterations), momentum 0.9 and weight decay 0.0001. During training, data augmentation was used to improve the robustness of the model against appearance variability of histology images (random horizontal flip, random hue rotation, random gamma correction, channel-wise random intensity shift). Over training (100 000 iterations), we saved the state of the model that minimized the cross-entropy in the corresponding validation partition. In a second stage, we froze the 53 first layers of the trained model from the first-stage and fine-tuned the last two layers in order to optimize slide-level classification performance. To this purpose, we pre-computed the vector output of the 53rd layer for all training image patches and re-trained the last two layers by using batches that include all the image patches of 16 random WSIs of the training partition with uniform distribution of transcriptional CMS. During this second training stage, all the predicted probability scores for all the image patches from the same WSI were averaged and the model was optimized via minimization of the cross-entropy loss for the averaged probability scores.

To assess the generalization of imCMS on unseen cohorts, we trained all models with data from a fixed development set, and systematically reported performance with test data from held out cohorts that were not used for training, fine-tuning or any part of the model selection procedure.

Statistical Analysis

Four logistic regression models for presence vs. lack of presence of each imCMS class were built for the endpoint of pCR. Models were adjusted by cohort (i.e., ARISTOTLE and SALZBURG) and the clinical confounders pretreatment T stage and pretreatment N stage, determined from pretreatment MRI assessments (i.e., at the time of clinical decision). The category ‘mixed imCMS’ was not analysed as it only contained two cases. p-values <0.05 were considered statistically significant. Statistical analyses were conducted using R [12].

SUPPLEMENTARY REFERENCES

[1] K. Sirinukunwattana, E. Domingo, S. Richman et al. Image-based consensus molecular subtype (imCMS) classification of colorectal cancer using deep learning. *Gut*, 70(3):544–554, 2021.

[2] L. Marisa, Y. Blum, J. Taieb et al. Intratumor CMS heterogeneity impacts patient prognosis in localized colon cancer. *Clinical Cancer Research*, 17:4768–4780, 2021.

[3] A. Valdeolivas, B. Amberg, N. Giroud et al. Charting the heterogeneity of colorectal cancer consensus molecular subtypes using spatial transcriptomics., 2023. [preprint] https://doi.org/10.1101/2023.01.23.525135.

[4] M. Seymour, T. Maughan, J. Ledermann et al. Different strategies of sequential and combination chemotherapy for patients with poor prognosis advanced colorectal cancer (MRC FOCUS): a randomised controlled trial. *The Lancet*, 370(9582):143–152, 2007.

[5] R. Adams, A. Meade, M. Seymour et al. Intermittent versus continuous oxaliplatin and fluoropyrimidine combination chemotherapy for first-line treatment of advanced colorectal cancer: results of the randomised phase 3 MRC COIN trial. *The Lancet Oncology*, 12(7):642–653, 2011.

[6] D. Sebag-Montefiore, R. Adams, S. Gollins et al. ARISTOTLE: a phase III trial comparing concurrent capecitabine with capecitabine and irinotecan (Ir) chemoradiation as preoperative treatment for MRI-defined locally advanced rectal cancer (LARC). *J Clin Oncol*, 38:4101–4101, 2020.

[7] Cancer Genome Atlas Network. Comprehensive molecular portraits of human breast tumours. *Nature*, 490(7418): 61–70, 2012.

[8] K. He, X. Zhang, S. Ren and J. Sun. Identity mappings in deep residual networks. In *European Conference on Computer Vision (ECCV)*, pages 630–645, 2016.

[9] M. Lafarge, E. Bekkers, J. Pluim et al. Roto-translation equivariant convolutional networks: Application to histopathology image analysis. *Medical Image Analysis*, 68:101849, 2021.

[10] S. Graham, D. Epstein and N. Rajpoot. Dense steerable filter CNNs for exploiting rotational symmetry in histology images. *IEEE Transactions on Medical Imaging*, 39.12:4124–4136, 2020.

[11] B. Veeling, J. Linmans, J. Winkens et al. Rotation equivariant CNNs for digital pathology. In *Proceedings of the International Conference on Medical Image Computing and Computer-Assisted Intervention (MICCAI)*, pages 210–218, 2018.

[12] R Foundation for Statistical Computing, Vienna, Austria. R: a language and environment for statistical computing., 2021. [url] https://www.R-project.org.
